# The UK national breast cancer screening programme for survivors of Hodgkin lymphoma detects breast cancer at an early stage

**DOI:** 10.1038/sj.bjc.6605215

**Published:** 2009-08-11

**Authors:** S J Howell, C Searle, V Goode, T Gardener, K Linton, R A Cowan, M A Harris, P Hopwood, R Swindell, A Norman, J Kennedy, A Howell, A M Wardley, J A Radford

**Affiliations:** 1 Department of Medical Oncology, The Christie NHS Foundation Trust, Wilmslow Road, Manchester, UK; 2 Department of Clinical Oncology, The Christie NHS Foundation Trust, Wilmslow Road, Manchester, UK; 3 Department of Psycho-oncology, The Christie NHS Foundation Trust, Wilmslow Road, Manchester, UK; 4 Department of Medical Statistics, School of Cancer and Imaging Sciences, The Christie NHS Foundation Trust, Wilmslow Road, Manchester, UK; 5 Department of Nursing and Governance, The Christie NHS Foundation Trust, Wilmslow Road, Manchester, UK; 6 Northwest Cancer Intelligence Service (formerly North West Cancer Registry), Kinnaird Road, Manchester, UK; 7 School of Cancer and Imaging Sciences, The University of Manchester, Oxford Road, Manchester, UK

**Keywords:** breast, cancer, Hodgkin, lymphoma, supradiaphragmatic, radiotherapy

## Abstract

**Background:**

Supradiaphragmatic radiotherapy (SRT) to treat Hodgkin's lymphoma (HL) at a young age increases the risk of breast cancer (BC). A national notification risk assessment and screening programme (NRASP) for women who were treated with SRT before the age of 36 years was instituted in the United Kingdom in 2003. In this study, we report the implementation and screening results from the largest English Cancer Network.

**Methods:**

A total of 417 eligible women were identified through cancer registry/hospital databases and from follow-up (FU) clinics. Screening results were collated retrospectively, and registry searches were used to capture BC cases.

**Results:**

Of the 417 women invited for clinical review, 243 (58%) attended. Of these 417 women, 23 (5.5%) have been diagnosed with BC, a standardised incidence ratio of 2.9 compared with the age-matched general population. Of five invasive BCs diagnosed within the NRASP, none involved axillary lymph nodes compared with 7 of 13 (54%) diagnosed outside the programme (*P*<0.10). The mean latency for BC cases was 19.5±8.35 years and the mean FU duration for those unaffected by BC was 14.6±9.11 years (*P*<0.01), suggesting that those unaffected by BC remain at high risk. Recall and negative biopsy rates were acceptable (10.5 and 0.8%, respectively).

**Conclusions:**

The NRASP appears to detect BC at an early stage with acceptable biopsy rates, although numbers are small. Determination of NRASP results on a national basis is required for the accurate evaluation of screening efficacy in women previously treated with SRT.

Treatment of Hodgkin lymphoma (HL) with supradiaphragmatic radiotherapy (SRT) at a young age increases the incidence of second cancers and non-malignant co-morbidities in later life (Greenfield *et al*, 2006). Cohort studies have shown the 25-year cumulative risk of breast cancer (BC) in women who were treated with SRT in childhood and young adulthood to be approximately 10–33%, compared with a lifetime risk in the female UK population of 11% (Department-of-Health, 2003; Taylor *et al*, 2007; Cooper and Westlake, 2008). The relative risk (RR) is greatest in women treated with SRT during adolescence and young adulthood, although an increase in risk is shown in most studies with SRT up to the age of 30–40 years, which persists for at least 20–25 years after treatment (Tucker *et al*, 1988; Hancock *et al*, 1993; Bhatia *et al*, 1996; Mauch *et al*, 1996; Aisenberg *et al*, 1997; Metayer *et al*, 2000; Swerdlow *et al*, 2000; Foss Abrahamsen *et al*, 2002; Ng *et al*, 2002; Travis *et al*, 2003; van Leeuwen *et al*, 2003; Taylor *et al*, 2007). The iatrogenic induction of a threefold increase in the RR of a common cancer, with major health, social and economic burdens, prompted the launch of a UK-wide national notification risk assessment and screening programme (NRASP) in November 2003 (Department-of-Health, 2003). In this study, we report the implementation and results of this exercise in the Greater Manchester and Cheshire Cancer Network (GMCCN) the largest Cancer Network in England serving a population of 3.2 million people.

## Materials and methods

### Patient identification and contact

The NRASP was launched in November 2003. In our centre, the North Western Cancer Registry (NWCR) and databases of The Christie (CHD; from 1965) were interrogated to identify eligible women residing in the GMCCN and/or managed at The Christie. An NWCR search was also completed of all patients within this cohort who had subsequently developed BC. Patients fulfilling all search criteria were entered into the National Strategic Tracing Service, and a letter was sent to their general practitioners (GPs) to verify their current status and contact details. Invitation letters were sent in November 2003, and again in March 2004 if no reply was received. A telephone help line to provide information about the NRASP, to answer queries and to facilitate an appropriate recall of patients was staffed by experienced nurses for 3 weeks.

### Screening plan

Screening protocols for this exercise were established and the rationale was subsequently published by a national expert committee (Table 1; Ralleigh and Given-Wilson (2004); Faulkner and Law (2005)). Screening was to begin in women who were ⩾8 years after SRT and at least 25-years-old, whichever occurred later. Five mammography and two magnetic resonance imaging (MRI) centres with sufficient capacity were identified within the GMCCN. It was agreed on a national basis that women would be screened by the network serving their current residence, even if treated for HL in a different network.

### Patient review

Patients were reviewed at routine HL follow-up (FU) clinics or at one of nine specially convened evening clinics. Consultations covered explanations of the risk and appropriate screening programme and, after informed consent, completion of a national questionnaire to capture data on risk factors for secondary cancers after HL treatment. Data were collected with regard to the dose and date of SRT, chemotherapy regimen, relapse status and diagnosis of other secondary malignancies. Simple cancer prevention and health education strategies, such as smoking cessation, were also discussed. An individual screening plan conforming to the national guidelines was constructed for each woman and communicated to their GP by letter (Table 1).

### Data collection

In total, 29 screening units were contacted in August 2007 to request screening results for all women who entered the NRASP in 2003, whether they were being screened annually or every 3 years as part of the National Health Breast Screening Programme (NHSBSP). For cases in which no results were forthcoming, hospital notes were searched and/or GPs were contacted for screening reports. The NWCR was interrogated for diagnoses of invasive and *in situ* breast carcinomas in the women eligible for inclusion in the study, and clinical notes of these patients were reviewed to extract clinicopathological data on their BC diagnoses and previous treatment details for HL.

### Statistical analysis

The expected number of BC cases in the study population was derived by multiplying each individual's years at risk by age-standardised crude incidence rates (of *in situ* and invasive BC). Incidence rates were available for the GMCCN from 1985 to 2005 and for England and Wales from 1967 to 1984. Data for 1963–1966 and for 2006–2008 were not available, and the annual rates for 1967 and 2005, respectively, were used for these periods. The standardised incidence rate (SIR) was expressed as the ratio of observed to expected number of BC cases. Mean ages and radiotherapy doses were compared using Student's test, with adjusted degrees of freedom for unequal variances wherever appropriate. Frequency proportions between two groups were analysed using 2 × 2 contingency tables using the *χ*^2^ test with Yates’ continuity correction or Fisher's exact test wherever appropriate. SPSS package (SPSS Woking, Surrey, UK) was used for these calculations.

## Results

### Patient identification and review

**Figure 1:**
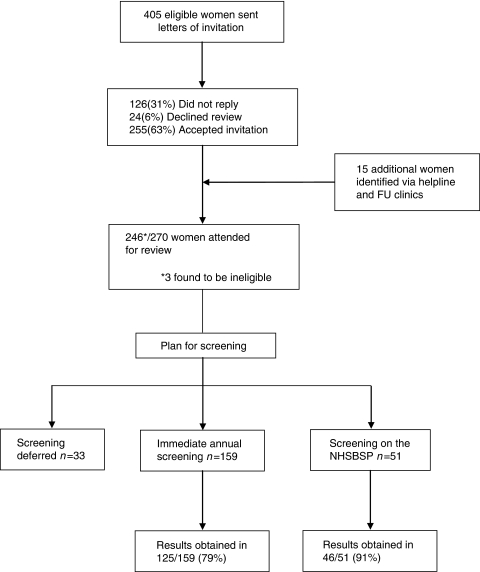
Flow diagram depicting the inclusion process for NRASP, the proportion of women requiring screening and the number of women for whom screening reports were available. FU, follow-up; NHSBSP, National Health Service Breast Screening Programme.

Database searches revealed 405 eligible women and a further 15 were identified through the telephone helpline or FU clinics, having received SRT at another institution but now residing in the GMCCN. The processing of these women is described in Figure 1 and, out of a total of 417 eligible patients, 243 (58%) were reviewed, counselled and referred for screening if required.

### Breast cancer risk

In the cohort of 417 women eligible for inclusion in the programme, NWCR searches have identified 23 (5.5%) with at least one diagnosis of breast malignancy. The expected number of cases of BC in this population up to 2008 was 8.0, thus the SIR for women treated with SRT for HL was 2.9. From the NRASP inception, the SIR of BC in women as yet unaffected was 3.75 (O 6.0 /E 1.6) in those reviewed and 2 (O 3/E 1.5) in those not reviewed. Table 2 presents the significant differences between these two patient populations with regard to age at SRT, attained age and years at risk of BC. Supradiaphragmatic radiotherapy field characteristics did not differ between the two groups.

The women diagnosed with BC were considered as one cohort, as no significant differences were found in the age at SRT, age at NRASP, SRT dose or SRT field between the group that was reviewed (*n*=12) and the group that was not (*n*=11; data not shown). However, those with BC had a significantly higher mean number of years for risk of BC (censored at first diagnosis of BC) and were more likely to have received a mantle field and a higher SRT dose than those without BC (Table 3).

Mantle field radiotherapy was delivered at a higher mean dose (3416±474.0) compared with all other fields combined (3018±707.5; *P*<0.0005). However, of the patients treated with mantle field, there was no significant difference in dose between those who did or did not develop BC (mean 3522±459.3 *vs* 3392±477.5; *P*=0.32). The proportion of women receiving a mantle field in each decade up to 2003 peaked at 70% during the period 1974–1983, falling to 14% from 1994 to 2003.

### Screening

Of the 243 eligible women who were reviewed, screening referrals are outlined in Figure 1. None of the women who required screening were aged <30 years and all were referred for mammography in accordance with the protocol. One such woman was screened with MRI alone because of local interpretation of the protocol, and her MRI was reported as normal. All other MRI scans were performed on young women to further evaluate mammographically normal but dense breasts, and were reported as normal. Of the 210 patients referred for screening, 9 (4.3%) subsequently declined and no evidence of screening could be found for 30 (14.3%) patients. At the time of writing, 370 screening results have been recovered for 171 of the 210 women (81%) in whom screening should have been initiated, that is, a mean of 2.2 screens per patient (Table 4).

So far, 39 of 370 (10.5%) screening episodes have resulted in patient recall, (Table 4) with 22 of 175 (12.6%) women being recalled from their first screens and 17 of 195 (8.9%) from second or subsequent screening episodes. In 31 of 39 screening episodes that led to a recall, further mammographic views, USS or MRI was considered adequate and these women remain BC free. Eight of the 39 women underwent fine needle aspiration or core biopsy, and malignancy was confirmed in 5, giving a benign biopsy rate of 0.8% (3 of 364).

### BC cases and tumour characteristics

The characteristics of each BC case, including both HL and BC diagnosis and treatment, are presented in Table 5. Cases 3 and 6 were diagnosed with metachronous contralateral primary BCs after March 2004, case 3b through the NHSBSP and case 6b by palpation of an asymptomatic breast lump at a BC FU appointment. The latter BC was also visible by mammography. These metachronous cancers are not included in subsequent analyses. Three of the nine cases (15, 16 and 17) were diagnosed with BC after the start of the NRASP but were not enrolled within it.

Five of the six women reviewed in the NRASP who developed BC were diagnosed through the programme (cases 19–23, Table 5). The sixth (case 18) had not attended the NHSBSP from the age 50 years, and presented symptomatically at age 56 years before her first screen in the NRASP had been performed. This cancer was also visible mammographically. Five of these six cases were invasive ductal carcinomas (IDCs, cases 18–22), none of which involved the axillary lymph nodes (ALNs). In contrast, in women with early BC and known ALN status diagnosed outside the NRASP, 7 of 13 (54%) involved ALNs (*P*=0.10). Three of the cases diagnosed within the NRASP were ‘triple negative’ for oestrogen and progesterone receptors, as well as for HER-2, and two were grade 3 tumours (Table 5). No significant differences were detected in the median BC grade, size or oestrogen receptor expression in those diagnosed within or outside the NRASP. The proportion of women with a screen-detected BC was significantly higher in women diagnosed after the inception of the NRASP (1 of 14 *vs* 6 of 9; *P*=0.01).

### Breast cancer follow-up

In the eligible cohort of 417 women, 14 cases of BC were identified as occurring before the inception of the NRASP, with a median FU of 7 years. During this time, three patients suffered local recurrence, two developed contralateral primary BC and one developed metastatic BC from which she died. Two other women with a diagnosis of BC are known to have died, one from another secondary cancer (non-HL) and one from chronic obstructive pulmonary disease. With a median FU of only 1 year, no recurrence of BC has been seen in the cases identified through the NRASP.

## Discussion

Through an assessment of the NRASP in the largest English cancer network (GMCCN) with a population of 3.2 million, we have confirmed that SRT at a young age increases the risk of BC (SIR 2.9). This SIR is probably an underestimate of the true risk, as we had no contact with 31% of eligible women. These women may have been diagnosed with BC outside the GMCCN and such cases would not have been identified by searches of the NWCR alone. Indeed, this is likely to be the case as the risk factors for the development of BC were increased in the group not reviewed and the recorded SIR was lower than expected (Table 2). However, the increased SIR of BC in the population reviewed in the NRASP may also be due to a reduction in lead time after inception of the screening programme. The impact of shortened lead time on BC incidence will only be addressed appropriately by an examination of the national annual BC incidence rates in the HL cohort over a more protracted period of time.

In our cohort of 417 women, 243 (58%) attended for risk assessment. In the only other Cancer Network to report implementation results in this programme (North Trent), the uptake rate was comparable at 64% (77 of 120) (Greenfield *et al*, 2006). Three similar recall studies conducted in North America have published recall rates of 32% (115 of 360) (Lee *et al*, 2008), 28% (47 of 167) (Kwong *et al*, 2008) and 54% (90 of 167) (Diller *et al*, 2002). These data demonstrate the difficulties inherent in this type of exercise in which patients are contacted retrospectively, and argue strongly for the prospective identification and counselling of such women at completion of treatments known to be associated with late toxicity.

The primary goal of breast screening is to reduce BC mortality through early detection. The sensitivity and specificity of the screening programme should be optimised to minimise the psychological and physical morbidity associated with false-positive diagnoses. The majority of retrospective data suggests that BC occurring after SRT is detectable by mammography in 80–100% of cases (Dershaw *et al*, 1992; Yahalom *et al*, 1992; Tardivon *et al*, 1999; Ralleigh and Given-Wilson, 2004). The degree of sensitivity in a relatively young population is believed to be due to a high prevalence of micro-calcification associated with abnormal mammograms. However, these data are limited by small sample sizes and absent age-matched controls. In the three prospective recall studies from North America, 27 of 28 (96%) of the BCs diagnosed were evident on mammography (Diller *et al*, 2002; Kwong *et al*, 2008; Lee *et al*, 2008). After the inception of the NRASP in the GMCCN, no interval cancers have been diagnosed, five of six women who consented to screening and developed BC were diagnosed by mammography and the sixth presented symptomatically with a mammographically detectable invasive cancer before routine screening was initiated. Furthermore, the five additional women diagnosed with BC during the NRASP (cases 3b, 6b and 15–17; Table 5) all had BCs visible on mammography, although three of five (cases 6b, 16 and 17) presented symptomatically. These data and the lack of ALN involvement in all of the BCs detected through the NRASP suggest that the screening strategy is appropriate, although the number of cases is small.

In contrast, Lee *et al* (2008) found that six of seven invasive cancers detected through their prospective screening clinic were detected clinically. Although all but one of the seven BCs were visible by mammography, four of the women had had mammograms reported as normal within the preceding 6–12 months, and all of these women had ALN involvement (Lee *et al*, 2008). This group is currently examining the role of screening on a 6-monthly basis, alternating between mammography and MRI (Lee *et al*, 2008). The American Cancer Society has recommended the use of MRI to screen women at high risk of BC after SRT for HL in the absence of data demonstrating superior efficacy over mammography in this specific population, although the potential risks of additional small doses of radiation through mammography must be considered (Saslow *et al*, 2007). In other high-risk populations, MRI has demonstrated a significant improvement in diagnostic sensitivity, but at the expense of reduced specificity with increased recall (10–12%) and benign biopsy rates (Kriege *et al*, 2004; Leach *et al*, 2005; Saslow *et al*, 2007). In this study, the recall rate for first screens was 12.8% compared with 17.2% (17 of 99) and 12.7% (10 of 79) in the two other studies in this setting (Diller *et al*, 2002; NHSBSP, 2007; Kwong *et al*, 2008). The rate is higher than that for NHSBSP (8.3% during 2005–2006), almost certainly because of the younger age of the HL-screening population (NHSBSP, 2007). Indeed, exclusion of the 10 cases who were all <50 years of age and who were recalled for a reassessment of normal dense breasts by further imaging alone results in a recall rate of 6.9% (12 of 175). The benign biopsy rate in our report, at 0.8% (3 of 364), was higher than that in the NHSBSP (0.2%), perhaps reflecting the knowledge of the increased RR of this population by reporting radiologists, or the relatively small patient numbers in this study. No open biopsies were performed. The benign biopsy rates in the two prospective screening studies that reported this parameter were 3.8 and 6.1%, respectively (Diller *et al*, 2002; NHSBSP, 2007; Kwong *et al*, 2008).

The risk of developing BC after treatment for HL is related to both SRT dose and field (Tinger *et al*, 1997; Hill *et al*, 2005). In our cohort, the increased SRT dose received by BC cases was primarily a reflection of the increased incidence of a mantle field rather than an independent effect of dose on BC development. At The Christie, as elsewhere, the use of a mantle field for the treatment of women requiring SRT for HL has declined over time. In a recent report, a switch from a 35-Gy mantle field to involved field radiotherapy (IFRT) at the same total dose reduced the estimated 20-year excess RR of BC by 63% (Hodgson *et al*, 2007). This model is consistent with meta-analysis data of clinical trials demonstrating an odds ratio of 3.25 (*P*=0.04) for the development of BC for women treated with extended field *vs* IFRT (Franklin *et al*, 2006). It may be possible to avoid radiotherapy altogether in a proportion of patients with both early and advanced stage HL, thus reducing the detrimental long-term sequelae of treatment for these individuals. The challenge is to identify with precision those patients in whom SRT is absolutely required for optimal disease control and those in whom SRT can be safely omitted (Meyer *et al*, 2005; Diehl, 2007; Eich *et al*, 2007; Picardi *et al*, 2007). Studies to determine the role of FDG-PET imaging in this process are underway and promising interim results have been reported (Radford *et al*, 2008).

The introduction of widespread screening in developed countries is in part responsible for the decrease in BC mortality observed over the last 15–20 years (Nystrom *et al*, 2002; Berry *et al*, 2005). Screening detects BC at an earlier stage, with lower rates of ALN involvement, perhaps the most important of all negative prognostic factors in BC (Rosen *et al*, 1989). However, screening may also detect indolent cancers with little capacity to impact negatively on health; moreover, the method of BC detection (screening *vs* symptomatic) has itself recently been recognised as a prognostic factor for BC recurrence (Joensuu *et al*, 2004; Shen *et al*, 2005). In this study, 18 women with IDC or ILC had a known ALN status. In all, 7 of 13 (54%) women outside the NRASP had an ALN involvement compared with none (0 of 5) of those reviewed within it. Thus, the NRASP strategy seems to be capable of detecting BC at an early clinical stage, whereas the range of immunophenotypes suggests that these tumours are not of a universally good prognosis. Although new radiotherapy techniques and treatment strategies have the potential to reduce the future burden of late effects, the population of women reviewed in the NRASP has a mean age approaching that at which BC was diagnosed in the 23 affected women, suggesting that there remains a significant cohort at an increased risk of BC (and other malignancies) in whom continued vigilance is required (Tinger *et al*, 1997; Hodgson *et al*, 2007).

## Conclusions

This study indicates the qualified success of the NRASP in the largest English Cancer Network. Although the detected cancers were ALN negative, numbers are small and a national summing of the results of the programme should be instituted to fully evaluate all aspects of the screening strategy.

## Figures and Tables

**Table 1:** Breast screening protocol for women treated with SRT for HL aged <36 years

**Attained age (years)**	**Recommended surveillance**
<25	No imaging
25–29	Annual MRI±ultrasound^a^
30–50	Annual mammogram±MRI/ultrasound^b^
>50	3 yearly mammography on the NHSBSP

HL=Hodgkin's lymphoma; MRI=magnetic resonance imaging; NHSBSP=National Health Service Breast Screening Programme; SRT=supradiaphragmatic radiotherapy.

a Ultrasound to be used to further evaluate areas of suspicion on MRI.

b MRI or ultrasound to be used to further evaluate areas of suspicion on mammography.

**Table 2:** HL treatment and breast cancer risk demographics of women reviewed in the NRASP and those not reviewed

	**Women reviewed in NRASP (*n*=237)**	**Women not reviewed in NRASP (*n*=166)**	**Significance value**
Age at SRT (years±s.d.)	25.5±5.28	23.4±5.92	*P*<0.0005
Number treated with SRT aged <25 years (%)	116 (49)	106 (64)	*P*=0.002
Age at NRASP (years±s.d.)	40.3±9.09	43.2±11.81	*P*=0.008
Average numbers of years at risk of BC (years±s.d.)	14.6±9.11	19.6±11.10	*P*<0.0001
SIR of breast cancer	3.75 (O 6.0/E 1.6)	2 (O 3/E 1.5)	ND

BC=breast cancer; HL=Hodgkin's lymphoma; ND=not determined; NRASP=notification risk assessment and screening programme; SRT=supradiaphragmatic radiotherapy.

**Table 3:** Age and HL treatment demographics for women reviewed in the NRASP and for those diagnosed with BC

	**Women reviewed in NRASP excluding BC cases *n*= 231**	**BC cases *n*=23**	**Significance value**
Mean age at SRT (years±s.d.)	25.6±5.25	23.4±5.08	*P*=0.06
Number <25 years at SRT (%)	113 (49)	15 (65)	
Mean age at NRASP (years±s.d.)^a^	40.1±9.11	45.3±8.66	*P*=0.01
Mean number of years at risk of BC at NRASP inception (±s.d.)^a^	14.6±9.11	19.5±8.35	*P*=0.01
			
*Number with SRT field (%)* b			
Mantle	83 (34)	16 (70)	
Mediastinal	139 (57)	5 (22)	
Other	21 (8)	2 (9)	*P*<0.0005^c^
			
Mean SRT dose (±s.d.) (cGy)^d^	3137 (±676.9)	3367 (±449)	*P*=0.005
Alkylating chemotherapy (%)^e^	188/219 (86)	17/23 (74)	*P*=0.23

BC=breast cancer; HL=Hodgkin's lymphoma; NRASP=notification risk assessment and screening programme; SRT=supradiaphragmatic radiotherapy.

a Censored at the time of diagnosis of BC.

b SRT field data not available for four patients.

c Comparing mantle field and others combined.

d SRT dose unknown in seven patients.

e Chemotherapy details not known for 12 patients.

**Table 4:** Results of screening tests performed as part of the NRASP (including those screens performed on the NHSBSP in women ⩾50 years)

**Screening finding**	**Number of screens (%)**
Normal screen	315 (86.5)
Dense breasts on mammography – USS/MRI	20 (5.5)
Abnormal – further imaging but no malignancy	21 (5.8)
Abnormal – FNA or core biopsy but no malignancy	3 (0.8)
Abnormal – DCIS confirmed	1 (0.3)
Abnormal – invasive malignancy confirmed	4 (1.1)
Total	364 (100)

DCIS=ductal carcinoma *in situ*; FNA=fine needle aspiration; MRI=magnetic resonance imaging; NHSBSP=National Health Service Breast Screening Programme; NRASP=notification risk assessment and screening programme; USS=ultrasound scan.

**Table 5:** Individual cases of breast cancer in women treated with SRT for HL

			**Radiotherapy**				**Pathology**			**Immuno-phenotype**			**BC treatment**	
**Case**	**BC Latency (years)**	**HL type and stage**	**Dose (cGy)**	**Field**	**SDBC**	**ALN**	**Subtype**	**Size (mm)**	**Grade**	**ER/PR**	**Her2**	**BC Rec**	**Chemo**	**RT**
1	25	NS 1a	3000	H+N	N	0/7	IDC	40	2	ND	ND	N	N	CW
2	12	NS?	4100	M+SCF	N	0/9	IDC	4	2	ND	ND	N	N	N
3a	16	NS 2b	3500	M	N	0/8	IDC	40	3	−/−	−	N	AC	N
3b	29				Y	0/10	IDC	15	3	−/−	−	N	N	N
4	14	NS 3a	3500	M	N	0/?	IDC	5	2	ND	ND	N	N	N
5	12	NS 2b	3000	Med+N	N	0/15	IDC	12	3	−/−	ND	Y	N	B
6a	5	NS 2a	3500	M	N	0/?	DCIS	NK	—	ND	ND	N	N	N
6b	14				N	0/7	DCIS	10	3	−/−	+	N	N	N
7	10	NS 2a	3500	M	N	1/7	IDC	22	2	+/+	−	Y	CMF	N
8	21	NS 2a	3500	M	N	6/14	IDC	39	3	+/-	+	Y	CMF	N
9	12	MC 3b	3500	M	N	2/2	IDC	45	2	+/-	ND	N	FEC	N
10	23	MC 4b	3000	Med+N	N	1/9	IDC	14	1	+/+	+	Y	N	N
11	31	NS 4a	2500	M	Y	0/14	IDC	6	3	+/+	ND	N	N	N
12	16	NS 1a	3500	M	N	0/?	Sarcoma	NK	ND	ND	ND	Y	N	N
13	32	NS 4b	3100	Med+N+Ax	N	ND	ILC	28^a^	2	+/+	ND	N	N	N
14	17	MC 1a	3500	M	N	10/17	IDC	24	2	+/+	+	N	FEC-D	N
15a	20	NS 2a	3000	N+Ax	Y	2/9	IDC	15	1	+/+	+	N	N	N
15b					Y		IDC	14	3	+/+	+			
15c					Y	1/1	IDC	8	2	+/+	+			
16	10	NS 4b	3000	Med	N	1/12	IDC	110^a^	2	−/−	−	N	XD	CW
17a	27	NS 3a	3000	M	N	ND	Mucoid	NK	NK	NK	NK	M	N	N
17b					N	ND	IDC	40^a^	3	−/−	−			
18	30	LP 1a	3000	M	N	0/12	IDC	33	3	−/−	−	N	E-CMF	CW
19	14	NS 2a	3500	M	Y	0/11	IDC	26	2	+/+	ND	N	N	CW
20	33	MC 2	3000	Med+N	Y	0/5	IDC	5	1	+/+	ND	N	N	N
21	33	NS 2b	4750	M	Y	0/1	IDC	17	2	−/−	−	NK	NK	NK
22	15	NS 2b	3500	M	Y	0/15	IDC	15	3	−/−	−	N	XD	N
23	20	NS 2a	3500	M	Y	0/4	DCIS	140	3	−/−	−	N	N	N

AC=adriamycin cyclophosphamide; ALN=axillary lymph node; Ax=axilla; B=breast; BC=breast cancer; Chemo=chemotherapy; CMF=cyclophosphamide methotrexate 5-fluorouracil; CW=chest wall; DCIS=ductal carcinoma *in situ*; ER=oestrogen receptor; FEC 5-fluorouracil epirubicin cyclophosphamide; H=head; Her2=human epidermal growth factor receptor 2; HL=Hodgkin lymphoma; IDC=invasive ductal carcinoma; ILC=invasive lobular carcinoma; LP=lymphocyte predominant; M=mantle; MC=mixed cellularity; Med=mediastinum; N=neck; NS=nodular sclerosing; ND=not determined; NK=not known; PR=progesterone receptor; Rec=recurrence; RT=radiotherapy; SCF=supraclavicular fossa; SDBC=screen-detected BC; SRT=supradiaphragmatic radiotherapy; XD=capecitabine docetaxel.

a Clinical measurement only.
